# Value of Jaborandi and Pilocarpine in Eye Diseases

**Published:** 1882-03

**Authors:** 


### ORIGINAL TRANSLATIONS AND ABSTRACTS.

### THE VALUE OF JABORANDI AND PILOCARPINE IN EYE DISEASES.

Dr. M. Landesberg, of Philadelphia, reports (*Med. and Surg. Reporter*, Feb. 4, 1882) his experience in the use of these remedies in various eye affections in which no other remedy but the jaborandi or pilocarpine was employed.

He used extract of jaborandi in ten cases of parenchymatous keratitis, with no effect whatever.

In two cases of panophthalmitis, with no effect upon the course of the morbid process, but some relief to the pain and abatement of the inflammatory symptoms. In seven cases of hemorrhage into the anterior chamber and vitreous, consequent upon a blow upon the eye, vision, which had been reduced to perception of light only, was completely restored within two weeks.

In three cases of irido-choroiditis, with opacities of the vitreous, restored the normal condition of the vitreous and improved vision very markedly.

In two cases of irido-choroiditis serosa, with opacities of the vitreous, perfect recovery in one case—very marked improvement in the other.

In one case of equatorial choroiditis with circumscribed choroidal atrophies, diffuse infiltration of the retina and opacities of the vitreous, vision was increased from the power to count fingers at 5 feet to 20-40 after fourteen doses of the jaborandi. In two cases of neuro-retinitis, the jaborandi was given with entire success in one case—partial success in the other.

In three cases of genuine atrophy of the optic disc, considerable improvement of vision followed the use of jaborandi for 3 to 4 weeks. In one case of retinitis from Bright's disease, a considerable degree of improvement followed.

In four cases of detachment of the retina, no effect followed the jaborandi in two cases, moderate improvement in one, and complete recovery in one.

In two cases of paralysis of the external muscle of the right eye, muriate of pilocarpine was injected subcutaneously with complete recovery.

One case of hemorrhage into the vitreous with the same result.

In one case of central retinitis, with large, floating opacities of the vitreous, almost complete recovery after thirteen hypodermatic injections.

In one case of large, dense opacities of the vitreous complete resorption of the opacities and restoration of vision. In one case of chorio-retinitis with opacities of vitreous, very great improvement followed the pilocarpine injections. In three cases of detachment of retina of long standing, the pilocarpine had no effect.

In two cases of recent detachment of retina, the improvement after the use of the remedy was considerable. In one case of hemorrhagic retinitis, the treatment was followed by very considerable improvement.

In one case of retinitis pigmentosa, a very favorable result was obtained.

In one case of double optic neuritis, the improvement was marked but not very great.

In two cases of genuine atrophy of the optic nerve, vision was considerably augmented by the remedy.

In one case of amaurosis of both eyes from scarlatinal uremia, the eyes became much better, and the general condition improved after the pilocarpine. In one case of chronic scleritis and irido-choroiditis, and opacities of the cornea, improvement followed the use of the remedy.

The following general remarks on administration are added by Dr. Landesberg:

"My experience in the use of jaborandi, gained in adults, totally failed when I began to try the remedy in children. The first child which I treated with jaborandi was a girl of seven years, who suffered from parenchymatous keratitis of both eyes. The mother was ordered to give at first thirty drops, and eventually to raise the dose to sixty drops. But even the latter dose failed to produce any effect. On two teaspoonfuls the skin became somewhat moist. A full benefit was only obtained by injection of four teaspoonfuls of the drug. Perspiration was copious for about an hour. Salivation was almost absent. There were no unpleasant after-symptoms whatever. In the other seven cases of parenchymatous keratitis, observed in children between seven and twelve years of age, I had also to recur to high doses of jaborandi in order to produce the desired effect. In four children between nine and twelve years the dose was four teaspoonfuls; in three children, between seven and nine years, five teaspoonfuls. Salivation was absent in three children, and but very slight in four. Perspiration was less copious than in adults, and of shorter duration. The ill after-effects were very slight. Vomiting occurred only once, in a child which had taken a large supper before the ingestion of jaborandi. Nausea occurred in two children. The inconvenience generally felt was pain in the abdomen.

The effective dose for subcutaneous injections of pilocarpine, in adults, varied from $\frac{1}{4}$ to $\frac{3}{4}$ of a grain. Salivation generally set in within two to ten minutes; in several cases, however, instantaneously—before I had time to withdraw the needle. Perspiration followed after about two to ten minutes. The duration of salivation was longer than perspiration; the latter kept on for an hour to two hours, the former continued for half an hour to an hour longer. The action of the drug began, in all instances, with increase in the pulse-rate and mostly with congestion to the head. The respiration was accelerated and in several cases difficult. In a few instances there was dyspnœa. This stage was the most unpleasant one to the patients. But these phenomena vanished as soon as salivary secretion made its appearance, and general easiness took place with the occurrence of perspiration. In the course of the process there was a slight fall in the temperature of the body; the patients felt chilly and they required warmer covering.

Of twenty-seven adults in whom I used subcutaneous injections of pilocarpine, the after-symptoms were very stormy in two. Vomiting and nausea occurred after every injection, and the other inconveniences were very severe. In five patients there were no unpleasant after-effects at all. In the other patient the ill after-effects were but slight, and easily borne. Slight gastric catarrh was generally a consequence of the treatment, but it was entirely wanting in a few instances.

Inflammation of the subcutaneous tissue around the point of injection occurred only to one lady patient. The inflammation set in after every injection, but it was very slight, and subsided either spontaneously or on warm poultices.

In children, I had to administer from half a grain to one grain of pilocarpine, in order to gain a full effect. Perspiration was copious; salivation but moderate. The after symptoms either very slight or totally wanting. The children had not the slightest aversion to the treatment.

Either preparation was administered two or three hours after a light supper had been taken, the patient being in bed, covered with a blanket. Jaborandi was given in warm tea, in doses which varied from fifty drops to four teaspoonfuls. Of forty-three adults whom I treated with jaborandi, the desired effect was produced with fifty drops in five; with one teaspoonful and a half in ten; with two teaspoonfuls in twenty; with three teaspoonfuls in five; and with four teaspoonfuls in three patients. The effect took place in about fifteen to thirty minutes after the ingestion of the drug, beginning with salivary secretion; perspiration followed within ten to thirty minutes afterward. In no instance a reverse condition was observed, and in no case the absence of either effect noted. Salivation lasted for about one hour and a half to two and three hours; perspiration subsided earlier. There was no ill effect whatever in ten patients; in seven patients the unpleasant after-symptoms were very considerable; in the others they were but slight.

